# Neurologic Urinary Incontinence, Lower Urinary Tract Symptoms and Sexual Dysfunctions in Multiple Sclerosis: Expert Opinions Based on the Review of Current Evidences

**DOI:** 10.3390/jcm11216572

**Published:** 2022-11-05

**Authors:** Riccardo Bientinesi, Filippo Gavi, Simone Coluzzi, Viviana Nociti, Monia Marturano, Emilio Sacco

**Affiliations:** 1 Department of Urology, Fondazione Policlinico Universitario A. Gemelli IRCCS, Università Cattolica del Sacro Cuore, 00168 Rome, Italy; 2 Department of Ageing, Neurosciences, Head, Neck and Orthopaedics Sciences, Institute of Neurology, Fondazione Policlinico Universitario A. Gemelli IRCCS, Università Cattolica del Sacro Cuore, 00168 Rome, Italy; 3 Department of Woman and Child Health and Public Health Sciences, Urogynecology, Fondazione Policlinico Universitario A. Gemelli IRCCS, Università Cattolica del Sacro Cuore, 00168 Rome, Italy

**Keywords:** multiple sclerosis, lower urinary tract symptoms, sexual dysfunctions

## Abstract

Objective: To resume each specialist’s role in the management of neurologic urinary tract symptoms (nLUTS) and sexual dysfunctions (SD) in patients suffering from multiple sclerosis (MS). Material and Methods: We asked a neurologist, a urologist and a gynecologist, experts on neuro-urology and sexual dysfunction at our hospital, to resume their role in the management of nLUTS and SD in MS patients based on the review of current evidence. PubMed was used to review literature with a focus on nLUTS and SD in MS patients. Conclusions: The difference in symptomatology in MS patients is very wide. The more the CNS is involved, the more the variations and severity of nLUTS is present. SD have numerous causes and should always be assessed. Urologists play the director’s role in evaluating and treating these patients. Neurologist should play an important role, they must evaluate the potential mutual interactions between disease manifestations of MS and their treatments. Additionally, gynecologists play an important information sharing role in the management of patients with multiple sclerosis.

## 1. Introduction

Multiple sclerosis (MS) is an autoimmune disease that affects the neurological system and can involve any part of the central nervous system (CNS). The estimated prevalence of MS worldwide is 250 per 100,000 people [1]. MS is usually diagnosed in young people (20–40 years) and more often in females. Further, 90% of MS patients report some types of lower urinary tract symptoms (LUTS) [2]. Moreover, the North American Research Committee on MS (NARCOMS) stated that 65% of MS patients experience one or more LUTS, including sexual disorders, with consequences also in everyday life and marital life [3]. There are three types of MS. Secondarily relapsing-remitting MS is the most common type (85%), characterized by the alternation between acute neurological symptoms and a period of improvement. Progressive MS usually occurs 1 to 2 decades after the relapsing-remitting type. The periods of improvement are less frequent and replaced by a gradual worsening of neurological symptoms. Primarily progressive MS (15% of MS patients) is characterized by progressive and constant neurological symptoms, in which there are no sudden symptoms variation [4,5]. LUTS in MS patients are variegated, ranging from urgency to urge urinary incontinence, incomplete bladder emptying and/or hesitancy. Due to the multifocal and diffuse involvement of the CNS, LUTS in MS patients are variegated and different in severity among MS patients. Furthermore, most MS patients stated that LUTS cause a moderate to severe impact on their quality of life (QoL) [6]. In addition, LUTS are a serious risk for upper urinary tract safety [7]. The most common LUTS in MS patients are urgency, frequency and neurogenic detrusor overactivity (NDO) [8].

The urologist has an essential role in treating MS patients. Optimal evaluation tools and proper management options are important to acquire a thorough knowledge of the disease progression. Risk stratification of MS patients based on LUTS impact on the upper urinary tract deterioration and on patients’ QoL should be done routinely. Anticholinergics are the first line treatment for symptoms of overactive bladder (OAB). Second line therapies are advised if conservative therapies fail to reduce the potential damage of the upper urinary tract or to relieve patient’s symptoms. Intradetrusor injections of BTXA are a good option for treating refractory cases. However, more studies are needed to assess neuromodulation, BTX and anticholinergics efficacy in MS patients. Diminished sexual function is often described in MS patients and it is associated with more severe disability, pain and depression. Sexual function should be assessed during the follow-up and treatment options should be offered [6].

## 2. The Neurologist’s Role

Despite the high prevalence of LUTS and sexual dysfunctions and their impact on QoL in MS patients, only few neurologists appear to assess bladder, bowel or sexual dysfunction with their patients. Due to its predominant position in the management of MS patients, the neurologist should always assess the presence of LUTS and refer the patient to a urologist when necessary. Moreover, they are the joining link in the interdisciplinary communication about the potential mutual interactions between disease manifestations of MS and their treatments.

Presence of LUTS and SD in MS patients should always be assessed through a complete anamnesis (urgency, frequency, nocturia, incontinence and emptying problems, bowel symptoms). Voiding diaries and questionnaires are useful tools to assess the bother of urological symptoms and the overall daily impact on QoL as well as SD, might be helpful [9].

Neurologists may consider interactions between treatments and disease manifestations not targeted by the treatment. For example, amantadine, usually used in MS patients to treat fatigue, has side effects like bladder problems and acute confusion due to the anticholinergic effect. The neurologist’s role in the diagnosis and treatment of urinary tract dysfunctions and sexual impairment in MS patients cannot be overestimated but, they could have an important role in identifying early patients who need to be evaluated by urologists. Moreover, with management and treatment of MS patients generally coordinated by neurologists, it seems reasonable that they can be the contact person to whom other physicians can report on the dose and timing of all installed treatments. To date, despite the importance of these aspects, there is need for a better definition of the neurologist’s role in the management of these conditions in MS patients, to plan properly and for early management of LUTS and sexual impairment.

## 3. The Urologist’s Role

**Figure 1:**
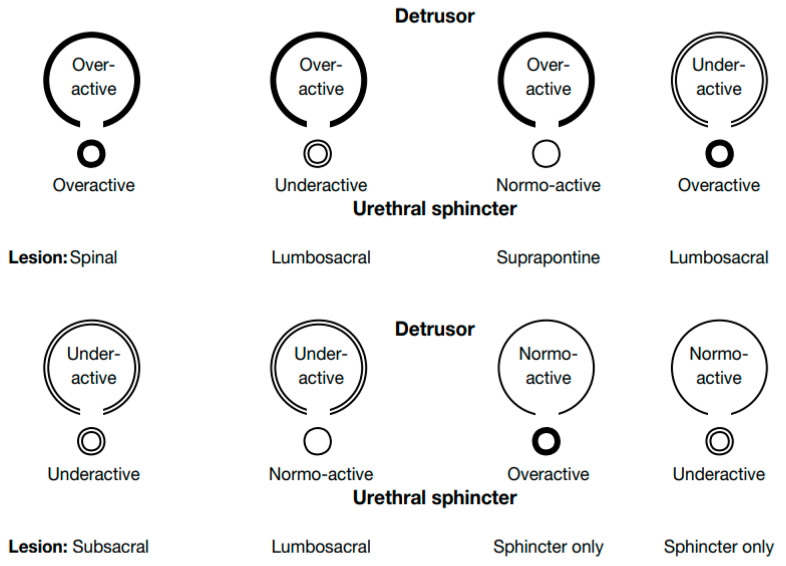
Madersbacher classification system showing typical neurological lesions.

Patients with MS can present lesions in any part of the CNS, although the spinal cord is most frequently affected. This explains the presence of neurogenic lower urinary tract dysfunction (NLUTD) in almost 80% of MS patients [10,11]. NLUTD usually occurs in advanced stages, but in 5 to 10% of MS patients can be seen in early stages [12]. NLUTD in MS patients can occur in several forms and severity depending on the vesicourethral behavior and symptomatology [13]. MS patients with incomplete spinal cord lesion usually have an alteration of the contraction of the bladder, which can be a combination of detrusor overactivity during the filling phase and a loss of the contraction during the voiding phase, with or without detrusor sphincter dyssynergia [14]. The literature suggests that NLUTD in MS patients is under-diagnosed (48% of patients) and up to 90% of patients are under-treated or do not receive optimal treatment [15]. To manage the dysfunction, to improve patient’s QoL and to avoid irreversible damage, an early diagnosis and treatment are essential. Once an early diagnosis has been made, identification of the different types of bladder dysfunction according to the level of the lesions can be useful, although in MS patients, spinal lesions can be found at different levels [16]. The NLUTD classification is of great importance, providing a standardized terminology and helping the urologist in dividing the patients into subgroups, depending on the neurological lesions and symptoms. Clinically it means that it would be possible to predict possible complications, guiding the urologist in the decision-making process during the follow up and treatment (e.g., in a young patient with overactive bladder and dyssynergia, it should be considered the possibility of bladder capacity reduction, resulting in possible ureteral refluxes due to high intravesical pressions. As a consequence of vesicoureteral reflux and hydronephrosis, renal function can be compromised. In a scenario like this one it would be essential during the follow up to always check renal function parameters, such as serum creatinine, and a renal echography could be helpful in excluding hydronephrosis and/or vesicoureteral reflux.) In the literature, there are several classifications. In 1990, Maderbacher redacted an accessible classification focusing on therapeutic consequences. Madersbarcher’s classification describes several NLUTD symptoms based on the contraction state of the bladder and external urethral sphincter during the voiding and filling phase (Figure 1) [17].

As we already said frequently, MS patients report bladder dysfunction. At least 80% of these patients reported some degree of LUTS according to the National MS Society, which is three times more common than the general population [18]. Common types of LUTS are hesitancy in starting urination, nocturia, urinary retention, increased frequency and/or urgency of urination and incontinence [8]. If not treated, these conditions can lead to worsening of other MS symptoms, such as urinary tract infections, ureteral reflux, kidney injuries and an important impact on QoL [19].

In 2020, a set of recommendations about LUTS in MS patients was generated and was published [20]. MS patients should be visited by a urologist if one or more of the following conditions occur: urinary incontinence, significant post-void residual volume (≥150 mL) with or without asymptomatic bacteriuria, quality of life impairment due to urological symptoms, recurrent urinary tract infections (UTI), an EDSS >3 or medical criteria. Moreover, bladder emptying efficiency is another factor to take in account when considering a referral to the urologist. In fact, we might find in clinical practice, patients with post-void residual volumes below 150 mL but a low emptying efficiency. A medical history, specific physical examination and a urodynamic study should aways be conducted in a MS patient with suspected NLUTD. Regarding the urodynamic study may not be performed in asymptomatic MS patients with normal free uroflowmetry and post-void residual, it should include cystometry, should consist of pressure-flow study and electromyography. No urodynamic study should be performed in case of MS flare, and it is necessary to wait for the stabilization of the patients’ symptoms.

MS patients with urological symptoms usually present other urological disorders: proststic hypertrophy in men, for example, and a differential diagnosis is recommended.

To know the disease process is essential to determine which therapy and management options are to be offered to each MS patient. LUTS should be assessed and treated with the goal of identifying early those patients at high risk of upper urinary tract deterioration an/or impaired quality of life. Anticholinergics are the first line treatment for OAB [20]. Second line therapies are advised if conservative therapies fail in reducing the risk of upper urinary tract deterioration or fail to mitigate patient’s symptoms. Intradetrusor injections of BTXA are a good option for treating refractory cases. However, more studies are needed to assess neuromodulation, BTX and anticholinergics efficacy in MS patients [21,22]. Neuromodulation as a treatment for neurogenic detrusor overactivity in MS patients has shown important results in the last decade and is an alternative to BTXA intradetrusor injections; however, still additional data are necessary to have clear clinical outcomes in such heterogeneous and complex patients [23].

Diminished sexual function is often described in MS patients, and it is associated with more severe disability, pain and depression. Sexual function should be assessed during the follow-up and treatment options should be offered [10]. Although the assessment of sexual dysfunctions (SD) in MS patients is often underestimated, they are usually present and they have an important impact on QoL [24,25]. It is essential not to forget that in the evaluation of sexual dysfunction, a complete clinical history should be taken and comorbidities such as diabetes, hypertension and obstructive sleep apnea, among others, should be considered [24]. Between 50–90% of MS patients reported to suffer from SD [8,26]. SD in MS patients are classified into primary, secondary or tertiary sources. Primary SD are a direct consequence of demyelinating lesions in the CNS (sensory paresthesia in the genitals, erectile and ejaculatory dysfunctions). Secondary SD include non-sexual physical changes that can affect the sexual response (asthenia, bladder and bowel dysfunction and pain). Tertiary SD refers to cultural and psychosocial issues affecting sexual satisfaction or performance (low self-esteem, demoralization and communications difficulties) [27]. Due to the coexistence of organic and nonorganic elements causing SD in MS patients, a detailed evaluation is often required. Early professional support counselling and treatment to overcome their difficulties should be a continuing process. Therapies should be regularly revised to adjust to the neurological condition and SD [28].

## 4. The Gynecologist’s Role

Experience, supported by the current literature, reveals that gynecologists play an important information sharing role in the management of patients with multiple sclerosis (MS).

Their expertise must involve a highly perceptive approach to the intimate sphere of the patient who is unlikely to express herself autonomously, but who often must cope with both an objectively evident disability status and the burden of the concern associated with her not knowing the underlying pathology. In fact, the MS patient needs the gynecologist not only to manage fertility and pregnancy, but also to share any concerns and assess any problems related to her sexual health and to evaluate the impact on her quality of life of any alterations in the intimate sphere. Often in pregnancy management, the role of the gynecologist is largely focused on containing anxiety, but in general, worries are expressed and shared by patients themselves. Sexuality, on the other hand, is a subject that tends to remain undisclosed unless the specialist takes it up first [27]. The fact that sexual health is fundamental to the quality of life of individuals and couples is confirmed and reported by many studies in literature. Thus, conditions affecting the nervous system, which governs the mechanisms of initiation, maintenance and control of sexual function, interfere significantly with the somatic, emotional and cognitive components that motivate sexual behavior [29]. Female sexual dysfunction is a disorder with several etiological components: anatomical, psycho-social and behavioral. It is characterized by a decrease in sexual desire and arousal, difficulty/inability to have an orgasm and/or pain during sexual intercourse [30]. Therefore, if sexual dysfunction (SD) is common in women, it is probably more common in women affected by MS, although still being underestimated. In fact, the lack of validated diagnostic tools makes it difficult to measure accurately the prevalence of SD and its resulting discomfort.

A 2000 study revealed that 43% of the female population is affected by some form of sexual dysfunction, preventing a woman from having satisfying intimate experiences [31]. This figure is significantly higher in patients with chronic diseases [32] and in women with MS (40–74%) [33]. Moreover, although several studies in the literature highlight the correlation between multiple sclerosis and sexual dysfunction, a solution to this problem, which is underestimated and poorly studied by medical professionals and usually not spontaneously reported by the patient because of feelings of embarrassment, is still to be sought [34].

A 2019 Italian study conducted on 306 women highlighted and confirmed that the prevalence of SD and sexual distress is higher in women with MS compared to healthy patients, and that age, disability and depressive symptoms are associated with the worsening of sexual dysfunction [35]. In female sexual function, desire, arousal, receptivity, orgasm and satisfaction are closely related and reinforce one another because of complex psychophysical interactions; therefore, the integrity of the motor and neurovegetative systems is necessary. This accounts for the enormous vulnerability of the sexual function in patients with MS because multiple sclerosis compromises this integrity [36]. Some studies have promoted a conceptualization of sexual disorders in MS based on their etiopathogenesis, thus distinguishing among primary, secondary and tertiary SD [30,37].

Primary sexual dysfunction would result from central nervous system alterations (demyelinating lesions and neuroaxonal loss) that characterize the underlying disease and directly modify the sexual response: genital sensory alterations, decreased libido, arousal, and orgasmic disorders, reduced vaginal lubrication. Secondary sexual dysfunction, on the other hand, would be the result of MS-related physical disabilities, which indirectly influence sexual response: hypertonic perineal muscles, spasticity, sphincter dysfunction, muscle weakness.

Finally, tertiary sexual dysfunction would be determined by the emotional impact of MS on patients, with the onset of mood disorders and depression that necessarily alter the sensory component. However, in multiple sclerosis, sexual dysfunction can occur even when there is no serious disability [38]. Therefore, it is necessary to promote a cultural change in both healthcare providers and patients to foster a thorough anamnestic investigation. Some studies, carried out by assessing the sexual concerns or impairments perceived by individuals with MS, have estimated that in women, disorders such as reduced or absent genital sensitivity, vaginal dryness, orgasmic disorders and loss of libido range from 34 to 85%. Furthermore, another element which should not be overlooked is the patient’s hormonal condition (genitourinary syndrome of menopause) and the impact of any therapy on sexual functioning. Several common medications used to treat multiple sclerosis can exacerbate sexual dysfunction. In particular, selective serotonin reuptake inhibitors induce additional sexual adverse effects, and with regard to women, anorgasmia and reduced libido [30].

Another subject on which medical literature has focused on is the impact that being in a couple relationship can have on sexual disorders and their perception. In fact, it has been observed that single status and/or partnered status, as well as the quality of the relationship with the partner, are among the most significant factors in predicting the inability to achieve a satisfactory sexual arousal: women without partners are at 10-fold greater risk of having arousal disorders [39]. Women with partner support reported a significant improvement in sexual satisfaction over time [40]. Therefore, counselling sessions tailored to the patient’s needs, together with an accurate history taking, are the most appropriate choice. Rediscovering one’s own body and that of the partner is an important step forward toward restoring intimacy: the couple must learn to live with MS, rediscover its sexual harmony and strike a new balance. Many people affected by MS have decided to turn to a Sexual Assistant, a new professional figure providing a sort of erotic support service aimed at helping disabled people discover their sexuality, in the broadest sense of the word, and their bodies, taking steps on the path toward building a stronger self-esteem. In 2013, in Italy, the “Promoting committee for the implementation and support of popular initiatives for sexual assistance” was founded. Its main purpose is to foster a change of the regulatory framework, thus making the legal recognition of this profession possible, and ensuring that disabled patients’ right to sexual, psycho-physical and emotional health is recognized and guaranteed. The International Consultation on Sexual Medicine has developed an updated algorithm for an accurate diagnostic assessment of sexual dysfunctions in men and women, with specific recommendations regarding the taking of sexual history [41]. As treatment and preventive strategies could enable a more effective management of SD, it is necessary to focus on these aspects of the disease in the counselling phase. This confirms how important it is to associate symptoms with organic as well as psycho-emotional-relational causes [42]. A recent 2021 study promoted a standardized approach to MS patients to ease communication between doctors and patients, showing the need for clinical management tools in the treatment of patients with sexual disorders [43]. All the above show that MS patient management demands a complex gynecological approach: the specialist must not only possess medical competences, but also be endowed with a high degree of sensitivity necessary to consider the patient’s emotional fragility and the specific alterations associated with the underlying neurological pathology so as to improve the patient’s health in the broadest and most comprehensive sense.

However, further studies are necessary to identify the most appropriate tools for the assessment of MS patients enduring sexual disorders.

## 5. Strengths and Limitations

The authors have years of experience in treating patients with multiple sclerosis. Each one of them reviewed the literature and resumed it with their clinical experiences. This paper is not a systematic literature review and this could be seen as a limitation of the study.

## 6. Conclusions

The difference in symptomatology in MS patients is very wide. The more the CNS is involved, the more the variations and severity of nLUTS is present. SD have numerous causes and should always be assessed. Urologists play the director’s role in evaluating and treating these patients. Neurologist should play an important role, they must evaluate the potential mutual interactions between disease manifestations of MS and their treatments. Additionally, gynecologists play an important information sharing role in the management of patients with multiple sclerosis.

## Data Availability

Not applicable.

## References

[1] Compston A., Coles A. (2002). Multiple sclerosis. Lancet.

[2] De Sèze M., Ruffion A., Denys P., Joseph P.A., Perrouin-Verbe B. (2007). International Francophone Neuro-Urological expert study group (GENULF). The neurogenic bladder in multiple sclerosis: Review of the literature and proposal of management guidelines. Mult. Scler. J..

[3] Phé V., Chartier–Kastler E., Panicker J.N. (2016). Management of neurogenic bladder in patients with multiple sclerosis. Nat. Rev. Urol..

[4] Mahajan S., Patel P., Marrie R.A. (2010). Under Treatment of Overactive Bladder Symptoms in Patients with Multiple Sclerosis: An Ancillary Analysis of the NARCOMS Patient Registry. J. Urol..

[5] Bientinesi R., Coluzzi S., Gavi F., Nociti V., Gandi C., Marino F., Moretto S., Mirabella M., Bassi P., Sacco E. (2022). The Impact of Neurogenic Lower Urinary Tract Symptoms and Erectile Dysfunctions on Marital Relationship in Men with Multiple Sclerosis: A Single Cohort Study. J. Clin. Med..

[6] Fowler C.J., Panicker J.N., Drake M., Harris C., Harrison S.C.W., Kirby M., Lucas M., MacLeod N., Mangnall J., North A. (2009). A UK consensus on the management of the bladder in multiple sclerosis. Postgrad. Med. J..

[7] Thompson A.J., Baranzini S.E., Geurts J., Hemmer B., Ciccarelli O. (2018). Multiple sclerosis. Lancet.

[8] Lublin F.D., Reingold S.C., Cohen J.A., Cutter G.R., Sørensen P.S. (2014). Defining the clinical course of multiple sclerosis. Neurology.

[9] Lemack G.E., Hawker K., Frohman E. (2005). Incidence of upper tract abnormalities in patients with neurovesical dysfunction secondary to multiple sclerosis: Analysis of risk factors at initial urologic evaluation. Urology.

[10] Bientinesi R., Gandi C., Bassi P. (2020). Managing Urological Disorders in Multiple Sclerosis Patients: A Review of Available and Emerging Therapies. Int. Neurourol. J..

[11] De Ridder D., Van der Aa F., Debruyne J., D’hooghe M.B., Dubois B., Guillaume D., Heerings M., Ilsbroukx S., Medaer R., Nagels G. (2013). Consensus guidelines on the neurologist’s role in the management of neurogenic lower urinary tract dysfunction in multiple sclerosis. Clin. Neurol. Neurosurg..

[12] Averbeck M.A., Iacovelli V., Panicker J., Schurch B., Agrò E.F. (2020). Urodynamics in patients with multiple sclerosis: A consensus statement from a urodynamic experts working group. Neurourol. Urodyn..

[13] Litwiller S.E., Frohman E.M., Zimmern P.E. (1999). Multiple sclerosis and the urologist. J. Urol..

[14] Panicker J.N., Fowler C.J., Kessler T.M. (2015). Lower urinary tract dysfunction in the neurological patient: Clinical assessment and management. Lancet Neurol..

[15] Bientinesi R., Campetella M., Nociti V., Bassi P., Sacco E. (2021). Identification of Brain Structures Involved in Lower Urinary Tract Symptoms and Sexual Dysfunctions in Patients with Multiple Sclerosis. Glob. J. Med. Res..

[16] Madersbacher H. (1990). The various types of neurogenic bladder dysfunction: An update of current therapeutic concepts. Spinal Cord.

[17] Minassian V.A., Drutz H.P., Al-Badr A. (2003). Urinary incontinence as a worldwide problem. Int. J. Gynecol. Obstet..

[18] Browne C., Salmon N., Kehoe M. (2015). Bladder dysfunction and quality of life for people with multiple sclerosis. Disabil. Rehabil..

[19] Medina-Polo J., Adot J.M., Allué M., Arlandis S., Blasco P., Casanova B., Matías-Guiu J., Madurga B., Meza-Murillo E., Müller-Arteaga C. (2020). Consensus document on the multidisciplinary management of neurogenic lower urinary tract dysfunction in patients with multiple sclerosis. Neurourol. Urodyn..

[20] Sacco E., Bientinesi R. (2015). Innovative pharmacotherapies for women with overactive bladder: Where are we now and what is in the pipeline?. Int. Urogynecol. J..

[21] Schmidt E.Z., Hofmann P., Niederwieser G., Kapfhammer H.-P., Bonelli R.M. (2005). Sexuality in multiple sclerosis. J. Neural Transm..

[22] Bientinesi R., Sacco E. (2018). Managing urinary incontinence in women—A review of new and emerging pharmacotherapy. Expert Opin. Pharmacother..

[23] van Ophoven A., Engelberg S., Lilley H., Sievert K.-D. (2021). Systematic Literature Review and Meta-Analysis of Sacral Neuromodulation (SNM) in Patients with Neurogenic Lower Urinary Tract Dysfunction (nLUTD): Over 20 Years’ Experience and Future Directions. Adv. Ther..

[24] Cantone E., Massanova M., Crocetto F., Barone B., Esposito F., Arcaniolo D., Corlianò F., Romano L., Motta G., Celia A. (2022). The relationship between obstructive sleep apnoea and erectile dysfunction: An underdiagnosed link? A prospective cross-sectional study. Andrologia.

[25] Tepavcevic D.K., Kostic J., Basuroski I.D., Stojsavljevic N., Pekmezovic T., Drulovic J. (2008). The impact of sexual dysfunction on the quality of life measured by MSQoL-54 in patients with multiple sclerosis. Mult. Scler. J..

[26] Foley F.W., LaRocca N.G., Sanders A.S., Zemon V. (2001). Rehabilitation of intimacy and sexual dysfunction in couples with multiple sclerosis. Mult. Scler. J..

[27] Kessler T.M., Fowler C.J., Panicker J.N. (2009). Sexual dysfunction in multiple sclerosis. Expert Rev. Neurother..

[28] Li V., Haslam C., Pakzad M., Brownlee W.J., Panicker J.N. (2020). A practical approach to assessing and managing sexual dysfunction in multiple sclerosis. Pract. Neurol..

[29] Basson R., Rees P., Wang R., Montejo A.L., Incrocci L. (2010). Sexual Function in Chronic Illness. J. Sex. Med..

[30] Salonia A., Munarriz R., Naspro R., Nappi R., Briganti A., Chionna R., Federghini F., Mirone V., Rigatti P., Goldstein I. (2004). Women’s sexual dysfunction: A pathophysiological review. Br. J. Urol..

[31] Rosen C., Brown J., Heiman S., Leib R. (2000). The Female Sexual Function Index (FSFI): A Multidimensional Self-Report Instrument for the Assessment of Female Sexual Function. J. Sex Marital. Ther..

[32] Hulter B.M., Lundberg O.P. (1995). Sexual function in women with advanced multiple sclerosis. J. Neurol. Neurosurg. Psychiatry.

[33] Alarcia-Alejos R., Ara-Callizo J.R., Martín-Martínez J., García-Gomara M.J. (2007). Sexual dysfunction management in multiple scle-rosis. Rev. Neurol..

[34] Zivadinov R., Zorzon M., Bosco A., Bragadin L.M., Moretti R., Bonfigli L., Iona L.G., Cazzato G. (1999). Sexual dysfunction in multiple sderosis: II. Correlation analysis. Mult. Scler. J..

[35] Gava G., Visconti M., Salvi F., Bartolomei I., Seracchioli R., Meriggiola M.C. (2019). Prevalence and Psychopathological Determinants of Sexual Dysfunction and Related Distress in Women with and Without Multiple Sclerosis. J. Sex. Med..

[36] Mazzariol C., Di Tonno F., Piazza N., Pianon C. (2010). Sexual Dysfunctions in Female with Neurological Disorders. Urol. J..

[37] Christopherson J.M., Moore K., Foley F.W., Warren K.G. (2006). A comparison of written materials vs. materials and counselling for women with sexual dysfunction and multiple sclerosis. J. Clin. Nurs..

[38] Demirkiran M., Sarica Y., Uguz S., Yerdelen D., Aslan K. (2006). Multiple sclerosis patients with and without sexual dysfunction: Are there any differences?. Mult. Scler. J..

[39] Borello-France D., Leng W., O’Leary M., Xavier M., Erickson J., Chancellor M.B., Cannon T.W. (2004). Bladder and sexual function among women with multiple sclerosis. Mult. Scler. J..

[40] Blackmore D.E., Hart S.L., Albiani J.J., Mohr D.C. (2011). Improvements in partner support predict sexual satisfaction among individuals with multiple sclerosis. Rehabil. Psychol..

[41] Hatzichristou D., Kirana P.-S., Banner L., Althof S.E., Lonnee-Hoffmann R., Dennerstein L., Rosen R.C. (2016). Diagnosing Sexual Dysfunction in Men and Women: Sexual History Taking and the Role of Symptom Scales and Questionnaires. J. Sex. Med..

[42] Drulovic J., Kisic-Tepavcevic D., Pekmezovic T. (2020). Epidemiology, diagnosis and management of sexual dysfunction in multiple sclerosis. Acta Neurol. Belg..

[43] Altmann P., Leithner K., Leutmezer F., Monschein T., Ponleitner M., Stattmann M., Rommer P.S., Zrzavy T., Zulehner G., Berek K. (2021). Sexuality and Multiple Sclerosis: Patient and Doctor Perspectives. J. Sex. Med..

